# Knowledge and Self-Assessment of Dental Injuries and Oral Health among Croatian Professional Water Polo Players: A Cross-Sectional Study

**DOI:** 10.3390/sports11110223

**Published:** 2023-11-13

**Authors:** Antonija Tadin, Josip Buzov

**Affiliations:** 1 Department of Restorative Dental Medicine and Endodontics, Study of Dental Medicine, University of Split School of Medicine, 21000 Split, Croatia; 2 Department of Maxillofacial Surgery, Clinical Hospital Centre Split, 21000 Split, Croatia

**Keywords:** dental injuries, knowledge, mouthguard, oral health, oral hygiene, water polo

## Abstract

Objectives/Aim: The purpose of this study was to evaluate oral health knowledge, and the incidence of self-reported dental injuries incidence, the frequency of mouthguard use, oral hygiene habits, and oral health status among professional water polo players. Materials and Methods: During the 2022/2023 season, 114 water polo players from the Croatian First League participated in a questionnaire-based online survey. The data collected included sociodemographic and professional attributes, oral health knowledge questions, dental injury experiences, and practices related to mouthguard use. Respondents also rated their own oral health status and oral hygiene habits. The data were analyzed using descriptive statistics and a regression analysis. Results: The respondents demonstrated inadequate knowledge of oral health, with a mean score of 6.4 ± 2.6 out of 12. Better knowledge correlated positively with older age (*p* ˂ 0.05) and consistent flossing (*p* = 0.014). Additionally, 27.2% (31 of 114) of reported dental injuries were related directly to incidents which occurred while participating in water polo. These injuries occurred more frequently during games (54.8%) and often involved the anterior maxillary incisors (71.0%) due to player contact (87.1%). Awareness of mouthguards was high (93.9%), whereas their actual use was low (7.0%) because 35.1% of respondents reported discomfort wearing them. The respondents’ self-assessments revealed widespread dental problems, including tooth sensitivity (13.3%), erosion (15.8%), calculus (28.1%), and pigmentation (7.9%). Conclusions: The research results indicate a lack of adequate knowledge among the respondents regarding oral health. This deficit was not associated with oral hygiene habits or oral cavity conditions. These findings highlight the disparities in oral health awareness and practices associated with water polo participation and emphasize the importance of education and prevention efforts.

## 1. Introduction

Oral health serves as a vital indicator of overall well-being and quality of life [1]. In contrast to the prevailing notion that athletes generally enjoy excellent health, extensive research has consistently shown that athletes often experience subpar oral health. The oral health challenges faced by athletes, including dental caries, periodontal diseases, orofacial injuries, and dental erosions, are often linked to their lifestyle choices, dietary preferences, health practices, health perceptions, and oral health knowledge [2,3,4,5]. Different studies have confirmed a higher frequencies of dental erosion, dentine hypersensitivity, calculus formation, and dental staining in swimmers (“swimmer’s mouth”) due to the acidic environment of most chlorinated swimming pools [5,6,7,8]. Given the evidence indicating the potential negative impact of poor oral health on overall health and athletic training and performance, it is imperative to promote regular dental checkups, healthy oral hygiene practices, proper nutrition, and injury-prevention behaviors among athletes [3,8,9].

Water polo is a water sport known for its physical contact and features a blend of sprint swimming, throwing, and combat elements which include jumps, changes in direction, and snatches. These dynamic and explosive actions require significant physical, mental, and technical–tactical efforts from athletes, as well as exceptional strength and endurance [10,11]. Consequently, the frequency of injuries in water polo competitions are notably higher compared to other water sports. The injuries reported include acute traumatic events such as contusions, lacerations, dislocations, sprains, and fractures. In addition, overuse injuries resulting from repetitive swimming and overhead throwing have also been observed [12,13]. Due to the physical nature of water polo, which involves contact among players and high ball velocities, the prevalence of injuries in this sport is high. Head and facial injuries are the most frequently reported injuries [13,14]. According to various studies, the incidence of injuries to the head and facial area resulting in contusions, lacerations, and fractures is approximately 0.57/player × year. Injuries to the orofacial region and teeth are also prevalent, with a reported range between 21% and 57.9% across players [13]. Additionally, injuries are significantly more serious during matches than during training [12]. It has been observed that center players are more prone to accidental blows to the head or face from other players’ elbows or fists. In contrast, goalkeepers are more susceptible to head injuries from contact with the ball [13].

The utilization of appropriately fitted mouthguards is the most critical factor in preventing orofacial injuries related to sports. Despite the advocacy of dental experts regarding the mandatory use of mouthguards in high-risk contact and collision sports, there still needs to be more awareness about their significance among athletes and their coaches [15]. According to several sports associations, the primary factors contributing to the non-usage of mouthguards are discomfort experienced while speaking, a perceived negative impact on performance, and the absence of a mandate to wear them [16]. The International Dental Federation has classified water polo as a medium-risk sport for dental injuries. Although it does not mandate using a mouthguard, the Federation recommends the use of a mouthguard during both matches and training [15,17].

Only a few studies have addressed the risk of dental and oral injuries and the use of mouthguards among water polo players. All of these studies confirmed a high frequency of dental injuries and insufficient knowledge and usage of mouthguards among players [18,19,20,21]. However, we could not find any studies that specifically examined oral health among water polo players and, as a result, the knowledge of this area remains limited. This study aimed to assess oral health knowledge, self-reported oral health and oral health behaviors, and the incidence of dental injuries and mouthguard usage among male water polo players in the Croatian First League.

## 2. Materials and Methods

In 2023, a cross-sectional convenience-sampling study was carried out at the Department of Restorative Dental Medicine and Endodontics, School of Medicine, University of Split. The study received approval from the Ethics Committee of the School of Medicine. The study took place during the 2022/2023 water polo season with the participation of ten clubs from the Republic of Croatia playing in the First Croatian Water Polo League and regional leagues. Participation in the study was entirely voluntary for the selected players, with all procedures conducted in accordance with the ethical principles outlined in the Declaration of Helsinki. The presentation of the results followed the reporting guidelines provided by the STROBE (Strengthening the Reporting of Observational Studies in Epidemiology) statement, ensuring transparency and consistency in reporting this observational study’s findings [22].

At the beginning of the study, all participants were informed of the research objectives and the possibility of withdrawal. Participation was voluntary, and minors and individuals unwilling to participate in the study or who were provided incomplete information were excluded. The study focused on 114 male water polo players from the active squad of the First Croatian Water Polo League. The minimum sample size required (n = 109) was derived from the total number of water polo players registered with the Croatian Water Polo Federation in 2022 (N ≈ 150), assuming a confidence level of 95%, a margin of error of 5%, and a response distribution of 50%.

The questionnaire based on the research objectives was designed following validated questionnaires from the relevant literature [19,20,21,23,24,25,26,27,28,29]. It was distributed to participants during their training sessions and games via social networks, using Google Forms.

The questionnaire consisted of 53 questions divided into five categories. The first section collected the sociodemographic and professional data of the respondents (Q1—age, Q2—education level, Q3—socio-economic status, Q4—playing position, Q5—years of playing water polo, and Q6—weekly playing hours). The second section included 12 questions (Q7–Q18) with three answer choices (yes/no/do not know), each with one correct answer (yes), assessing oral health knowledge. The total knowledge score was calculated as the sum of correct answers, with a maximum achievable score of 12. According to Bloom’s classification, the participants’ overall knowledge was divided into three categories: good if the score ranged from 80% to 100% (9.6–12 points), moderate if it fell between 60% and 79% (9.5–7.3 points), and poor if it was below 60% (<7.2 points) [30]. The third section addressed questions about the frequency of dental injuries, the use of dental mouthguards, and related information among respondents (Q19–Q31). The fourth (Q34–Q40) and fifth sections (Q41–Q46) covered the oral hygiene behaviors and daily practices adopted by the respondents to uphold their oral health. These sections also explored the participants’ self-evaluations of their oral health status. The concluding set of questions centered around the perceived significance of knowledge regarding oral hygiene, overall health, and dental injuries (Q47–Q53).

All questions for this survey were reviewed and selected by two dentists and a dental student who was a water polo player. Before the distribution of the survey, a pilot study was conducted with ten former water polo players to ensure the transparency and legitimacy of the survey. The pilot study subjects were not included in the primary data. The internal consistency of the total scores of oral health knowledge yielded a Cronbach’s alpha coefficient of 0.687.

A data analysis was conducted using the Statistical Package for the Social Sciences, version 25 (SPSS, IBM Corp, Armonk, New York, NY, USA). The normality of the response distribution was assessed through the Kolmogorov–Smirnov test. For categorical data, a descriptive analysis was employed to calculate frequency and percentage, while quantitative data were presented as either the median and interquartile range (IQR) or the mean and standard deviation. Furthermore, a multiple linear model (GLM) analysis was performed to identify independent variables associated with higher scores in oral health knowledge, with sociodemographic, oral health and oral hygiene practice characteristics as predictors. Oral health knowledge served as the dependent variable in this analysis. The statistical significance level was set at *p* < 0.05.

## 3. Results

Table 1 shows the demographic and occupational characteristics of the respondents in relation to their general knowledge of oral health. The mean age of the respondents was 23.9 ± 4.8 years (range: 18 to 38, Md = 24, IQR = 10.0–26.0). On average, they had been practicing water polo for 13.7 ± 4.9 years (range: 5 to 30, Md = 14.0, IQR = 10.0–16.0) and actively playing the sport for 12.9 ± 6.1 years (range: 5 to 32, Md = 10, IQR = 8.0–18.0). Considering socio-demographic and professional characteristics as independent variables, it was determined that a higher level of oral health knowledge was linked to an older age of the respondents (*p* < 0.05). Contrarily, a lower level of knowledge was associated with a higher number of hours of training per week (*p* = 0.006).

The responses in Table 2 show that the respondents have inadequate knowledge about the factors that negatively affect oral health. On average, their knowledge was rated 6.4 ± 2.6 (Md = 6; IQR = 4.75–8.0) out of a possible 12 points (minimum 0; maximum 12). Approximately 40% of the respondents possessed knowledge levels that fell below the median. Only 43.0% did know that sports drinks and energy drinks can damage teeth by causing erosion, while only 21.9% of them knew that rinsing the mouth with fluoridated liquid after swimming can prevent erosion. Responses associated with knowledge of dental trauma similarly demonstrated suboptimal results. Only 19.3% knew that a permanent tooth knocked out by dental trauma could be reinserted into the oral cavity. Remarkably, 78.1% did not know that the knocked-out tooth should be held by its crown during reinsertion.

Table 3 shows the experiences of respondents regarding dental injuries and the use of protective devices. In total, 27.2% (31 out of 114) of the respondents reported sustaining a dental injury during their participation in water polo. Most of these injuries (54.8%) occurred during games, and about 25.8% occurred during training sessions. Among the participants who suffered a tooth injury while playing water polo, the anterior maxillary incisors were the most commonly affected teeth (71.0%), with contact with another player being the most frequent cause of tooth injuries (87.1%). The most common types of injuries were crown fractures (35.5%), tooth displacement within the jaw (22.6%), and tooth avulsion (12.9%). After experiencing a dental injury, a significant proportion of respondents sought dental help, with 80.6% of them consulting a dentist. The overwhelming majority of respondents (93.9%) were familiar with the purpose of dental mouthguards, and more than 50% of them believed that this protection helped prevent sports injuries. However, only 7.0% of the respondents reported that they use mouthguards. The main reason given for not using them was discomfort (35.1%).

Table 4 presents data on the participants’ self-assessment of their oral health. The majority of respondents reported no black-brown pigment (92.1%), no tartar on the anterior teeth (71.9%), no eroded enamel (84.2%), and no inflamed gums (86.0%). In addition, a large proportion of respondents reported having dental caries (30.7%). Merely 20.2% of respondents consistently attend semi-annual regular dental checkups. Considering the independent factors related to self-assessed oral health, it was determined that a higher level of knowledge about oral health is not associated with any independent oral health variables (*p* > 0.05). 

Table 5 shows the daily oral hygiene habits of the participants. Almost all of them use a toothbrush and toothpaste daily (97.4%). In addition, 33.3% use dental floss and 22.8% use an interdental brush in their routine. After analyzing the characteristics of the participants in relation to daily oral hygiene habits, it was found that higher theoretical knowledge of oral health positively correlates with daily flossing (*p* = 0.014).

Table 6 shows the responses to the respondents’ self-assessment of their knowledge about oral health. Over 50% of respondents rated their knowledge of oral hygiene as excellent or very good, and 55% of respondents had received oral health information from their chosen dentist.

## 4. Discussion

The study revealed inadequate oral health knowledge and education as suboptimal oral hygiene habits among water polo players. For example, 66.7% of the participants did not know that chlorinated water harms teeth, causing the erosion of enamel, and only 21.9% recognized the protective effects of fluoride mouthwashes. In addition, only 43.0% of the participants were aware of the risks of tooth erosion from sports and energy drinks. Most professional and sociodemographic factors had no effect on the respondents’ oral health knowledge, except that younger participant showed significantly less knowledge than older groups.

Water polo is a contact sport in which players physically interact to prevent the opposing team from winning [21]. The World Dental Organization classifies water polo as a moderate-risk sport for dental injuries [14]. The primary causes of dental injuries include collisions with teammates and head impacts from the ball [18]. Crown fractures and avulsions are the most common dental traumas [17]. In this study, 27.2% of the respondents reported having suffered dental injuries. Specifically, 70.9% of respondents with dental injuries (22 of 31) confirmed that maxillary incisors were the most commonly affected teeth in water-polo-related incidents. A comparison between water polo and handball, another team contact sport with similar tactics but different environments (water vs. ground), provides an interesting perspective. Both sports have an intermediate risk of dental injury according to the World Dental Organization [11,12,13,14]. A Swiss and German study of 112 handball players found that 10.0% sustained oral trauma [24]. The incidence of oral trauma in water polo players was significantly higher (70.9%) than in the handball study (10.0%). The results of this study show that 80.6% of the injured respondents (25 of 31) suffered trauma during the game, often involving player contact, which is consistent with the results of studies from Switzerland and Spain [17,21,25]. Impressively, 80.6% of the respondents who had sustained oral trauma immediately sought dental care, indicating a proactive approach to dental care. Although water polo players spend less time playing official games than training, most injuries occur during the games because of increased competition, which increases the risk of injuries [26]. Regarding knowledge of dental injury recovery procedures, 64.9% of the respondents rated their knowledge as good or very good. However, only 19.3% knew that a permanent tooth knocked out by dental trauma could be reinserted into the oral cavity. Similarly, 21.9% knew that the knocked-out tooth should be held by the crown during reinsertion. Interestingly, in a Swiss and German study of handball players, weaker knowledge prevailed, with only 8.9% of the 112 participants knowing that a knocked-out tooth can be reinserted [24]. 

Dental mouthguards play a pivotal role in sports by safeguarding teeth and preventing trauma [27,28]. A 2007 meta-analysis scrutinized mouthguard efficacy in curbing dental injuries, revealing a 1.6–1.9 risk reduction factor. Dental shields disperse impact forces, mitigating transmission to the dentition [27]. In our study, 93.9% of participants were acquainted with mouthguards. While 58.8% believed mouthguards are valuable for injury prevention in water polo, only 7.0% wore them during training or matches, with five of them (out of 114) favoring custom-made options. Discomfort was the main reason for the low usage of mouthguards. These findings are consistent with a 2012 Swiss survey in which only 7.7% of 415 respondents reported wearing a mouthguard [21]. Similarly, in the aforementioned handball study, only 8.0% of respondents used a mouthguard during training [24]. In a Croatian study of 229 athletes from the sports of taekwondo, karate, handball, and water polo, only 41.0% reported using mouthguards in spite of their effectiveness at preventing dental injuries. Of note, there were significant differences in mouthguard use between sports, with taekwondo (73.7%) and karate (70.7%) athletes showing higher use than handball (14.5%) and water polo (5.1%) players [19]. These differences highlight the multiple factors that influence the use of mouthguards in different sports [19,24,28,29]. In this study the majority of recommendations regarding mouthguard use came from dentists or trainers. The promotion of mouthguard usage is of the utmost importance in preventing oral injuries among athletes, and it entails critical roles for both dentists and coaches [15,16]. Notably, our findings contrast with the results of a study conducted among water polo coaches in Croatia in which nearly 70% of coaches reported recommending mouthguards for their players [31].

The majority of respondents in this study reported no effects on tooth enamel associated with chlorinated swimming pool water, such as black-brown pigment (92.1%), calcification (71.9%), and erosion (84.2%). A phenomenon known as “swimmer’s mouth” is characterized by hard, brown tartar deposits commonly found on the front teeth and occurring on a 1 μm thick enamel layer. This discoloration occurs when the higher pH of swimming pool water compared to saliva interacts with salivary proteins, rapidly breaking them down and resulting in organic deposits on swimmers’ teeth [5,6,7,8]. Professional swimmers who spend more than six hours a week in chemically treated water experience this. In contrast to a University of New Mexico study in which all participants reported tooth discoloration and/or black stains, only 7.9% of participants in our study reported such changes. In addition, 15.8% felt that their enamel was thinning or eroding. A University of New Mexico study linked pool chlorination to dental erosion, calculus, and discoloration in swimmers [8]. Daily swimming, especially for professional swimmers who spend many hours in swimming pools, constantly exposes teeth to a significant amount of chlorinated water. Prolonged contact with such water can cause discoloration and erosion of the teeth. The interaction between saliva and disinfectants in the swimming pool favors discoloration and erosion, which occur mainly on the buccal or lingual surfaces of the upper and lower incisors. Dental aesthetics in particular often bear the brunt of these discolorations, which can have a negative impact on psychological well-being [5,6,7,8,32]. In a study conducted in Castellon, Spain, swimmers from different clubs were found to have dark brown stains on the labial surfaces of the lower incisors. To mitigate such conditions, athletes’ awareness of potential risks can be increased through education in conjunction with regular dental examinations [32]. Only one-third of the respondents in this study linked chlorinated water with dental erosion. In addition, less than 25% of the participants were knowledgeable about preventing erosion by rinsing the mouth with water or fluoridated liquid after swimming pool exposure. These findings emphasize the significance of increasing awareness concerning oral health risks linked to swimming and advocating for the adoption of suitable preventive measures.

Through survey questions addressing oral health and hygiene habits, our aim was to establish whether our research results correlate with findings among athletes in other countries [3,23,33,34]. Notably, 69.3% of the respondents reported no cavities, while 86.0% claimed to be free of inflamed gums. Similarly, a comparable percentage reported no issues of tooth sensitivity or bleeding gums. These outcomes indicate a relatively healthier oral state among Croatian water polo players compared to athletes from other nations. Research among elite athletes has consistently highlighted a significant prevalence of gingivitis, tooth erosion, and caries [23]. Pioneering research conducted during the 1968 Olympics unveiled subpar oral health among diverse professional athletes [23]. Subsequent investigations involving participants from the 2012 London Olympics indicated that 75.8% exhibited gingivitis, 45.1% exhibited dental erosion, and 54.9% had caries [3]. Recent studies of British professional soccer players reiterated the concerning state of oral health. The research noted that 53.2% suffered from tooth erosion, 37.0% had untreated cavities, and 4.8% were affected by periodontitis [3]. These findings accentuate the pressing need for comprehensive oral health care within the athletic community.

Approximately 80% of the participants in this study recognized a close connection between their overall well-being and quality of life and oral health. This conflicts with a survey conducted during the 2012 Olympics in which only 30.2% saw a link between oral health and quality of life and nearly 20% believed that oral health influenced their training or performance [3] Regarding oral hygiene, 92.1% of the respondents reported using a toothbrush and toothpaste daily. The daily use of interdental brushes was reported by 22.8%, while flossing and mouth rinses were used by 33.3% and 30.7%, respectively. In contrast, a 2019 study of 647 athletes in Rio de Janeiro found that only 37.56% brushed their teeth several times a day, and 32.92% did not floss at all [35].

The strength of this study lies in the considerable number of participants from the first water polo league in Croatia (with a response rate of about 75.0%). Nevertheless, some limitations should be noted. In addition to being a cross-sectional observational study, another potential source of methodological bias is convenience sampling, which depended on the availability of participants and their voluntary consent. Since a self-report questionnaire was used, response bias also might have influenced the results. Some respondents may have found the questionnaire too long, possibly leading to disinterested responses in the later sections. In addition, the cross-sectional nature of the study makes it impossible to establish causal relationships or examine behavior longitudinally. It would also be desirable to conduct a clinical examination among the subjects so that their own self-report of oral health could be verified clinically. Future research should examine additional factors that shed light on the interplay between oral health knowledge and athletes’ general and oral well-being. In addition, the use of clinical examinations in lieu of self-reporting could improve the accuracy of this research. Also, in light of the information presented above, it is advisable to conduct additional research involving diverse groups of water sports athletes. This will enhance our understanding of potential variations in the variables under study and contribute to a more comprehensive knowledge of oral health and exercise habits.

## 5. Conclusions

The results of the study show that the knowledge about oral health in the group of water polo players is insufficient. The only factor that had an impact on knowledge was the advanced age of the respondents. While self-reported oral hygiene practices and oral health had a negligible effect on knowledge, an exception was observed in those who included flossing in their daily oral hygiene routine and showed better knowledge. Since most respondents emphasized the importance of oral health knowledge and its central role in promoting healthy behaviors, comprehensive education of the surveyed population is warranted. This approach should prioritize oral health education, with a particular emphasis on dental injuries.

## Figures and Tables

**Table 1:** Generalized linear model analysis of predictors (sociodemographic and professional characteristics) for a higher total oral health knowledge score.

Characteristics		n (%)	β (95% CI)	*p* Values
Age group	18–24 years	35 (30.7)	References	
	25–30 years	40 (35.1)	1.304 (0.368–2.311)	0.007 *
	≥31 years	39 (34.2)	1.253 (0.296–2.209)	0.010 *
Education level	High school	54 (47.4)	References	
	College	33 (28.9)	−0.077 (−1.286–1.132)	0.900
	University	27 (23.7)	0.128 (−1.118–1.375)	0.840
Socioeconomic status	Average	90 (78.9)	References	
	Above average	24 (21.9)	−0.850 (−1.981–0.280)	0.140
Player positions	Wing	35 (30.7)	References	
	Centre forwards	17 (14.9)	0.401 (−1.027–1.829)	0.582
	Centre back	17 (14.9)	−0.580 (−1.962–0.802)	0.441
	Flat	35 (30.7)	0.506 (−0.585–1.597)	0.364
	Goalkeeper	10 (8.8)	0.994 (−0.865–2.752)	0.308
Weekly training	˂10 h/week	62 (54.4)	References	
	10 do 15 h/week	17 (14.9)	−0.437 (−1.592–0.719)	0.459
	>15 h/week	35 (30.7)	−1.224 (−2.103–−0.345)	0.006 *
Training experience	˂10 years	24 (21.1)	References	
	10 do 15 years	55 (48.2)	−0.069 (−1.084–0.947)	0.894
	>15 years	35 (30.7)	0.519 (−0.622–1.660)	0.373

Data are presented as numbers (percentages). Reference knowledge category is “low”. β, beta coefficient; 95% CI, 95% confidence interval; * *p* ˂ 0.05.

**Table 2:** Frequency and proportion of correct responses to oral health knowledge index items.

Question	n (%)
Oral health is closely related to general health (Yes).	91 (79.8)
Oral health is closely related to quality of life (Yes).	92 (80.7)
The most common oral diseases are dental caries (tooth decay), periodontal diseases, and oral cancers (Yes).	82 (71.9)
Poor oral hygiene can lead to the development of dental caries and periodontitis (Yes).	101 (88.6)
Diet affects the development of dental caries, periodontitis, and oral cancer (Yes).	78 (68.4)
Sports drinks and energy drinks, due to their sugar and acid content, have the potential to erode teeth, damaging their surfaces (Yes).	49 (43.0)
Exposure to chlorinated water has the potential to lead to enamel erosion on teeth (Yes).	38 (33.3)
Dental erosion can be prevented by rinsing the mouth with water or fluoridated mouthwash after spending time in the pool, which effectively reduces the softening of the enamel (Yes).	25 (21.9)
Mouthguards are an effective way to prevent dental injuries during sports activities (Yes).	78 (68.4)
Knocked-out permanent teeth due to trauma can be replanted into the oral cavity (Yes).	22 (19.3)
A knocked-out tooth can be replanted by holding onto the crown, the part that is normally visible in the mouth (Yes).	25 (21.9)
The upper anterior teeth are particularly susceptible to dental trauma and are frequently affected by such incidents (Yes).	51 (44.7)

Data are presented as numbers (percentages).

**Table 3:** Frequencies of dental injuries and the use of a mouthguard among respondents.

Characteristics		n (%)
Experienced dental injuries	Yes	31 (27.2)
	No	83 (72.8)
Time of injury occurrence	Training	8 (7.0)
	Match	17 (14.9)
	Training and match	6 (5.3)
	Did not experience	83 (72.8)
Causes of injury	Contact with another player	27 (23.7)
	Contact with the ball	2 (1.8)
	Shot on goal	2 (1.8)
	Did not experience	83 (72.8)
Injured tooth	Upper front	22 (19.3)
	Upper back	4 (3.5)
	Lower front	5 (4.4)
	Lower back	0 (0)
	Did not experience	83 (72.8)
Type of tooth injury	Avulsion	4 (3.5)
	Crown fracture	11 (9.6)
	Tooth dislocation	7 (6.1)
	Other	9 (7.9)
	Did not experience	83 (72.8)
Sought dental help	Yes	25 (21.9)
	No	6 (5.3)
	Did not experience	83 (72.8)
Knowledge of mouthguard	Yes	107 (93.9)
	No	7 (6.1)
Use of mouthguard	Yes	8 (7.0)
	No	106 (93.0)
Type of mouthguard utilized	Stock	2 (1.7)
	Custom made	5 (4.4)
	Mouth formed	1 (0.9)
	Do not use it	106 (93.0)
Source of mouthguard recommendation	Dentist	5 (4.4)
	Coach	2 (1.8)
	Media	1 (0.9)
	Do not use it	106 (93.0)
Time of mouthguard usage	Training	2 (1.8)
	Match	3 (2.6)
	Training and match	3 (2.6)
	Do not use it	106 (93.0)
Reasons for not using mouthguards	Discomfort	40 (35.1)
	Not useful	11 (9.6)
	I couldn’t get it	3 (2.6)
	Aesthetic concerns	3 (2.6)
	Other	57 (50.0)
Utility of mouthguards for injury prevention	Yes	67 (58.8)
	No	12 (10.5)
	Do not know	35 (30.7)

Data are presented as numbers (percentages).

**Table 4:** Generalized linear model analysis of predictors (self-assessed oral health characteristics) for a higher total oral health knowledge score.

Characteristics		n (%)	β (95% CI)	*p* Values
Dental injury	Yes	31 (27.2)	0.285 (−0.648–1.217)	0.549
	No	83 (72.8)	Reference	
Yellowish-brown or dark-brown stains	Yes	9 (7.9)	0.631 (−1.073–2.336)	0.468
	No	105 (92.1)	Reference	
Dental tartar (calculus)	Yes	32 (28.1)	0.389 (−0.630–1.048)	0.455
	No	82 (71.9)	Reference	
Dental erosion	Yes	18 (15.8)	0.163 (−1.135–1.460)	0.806
	No	95 (84.2)	Reference	
Inflamed gums	Yes	16 (14.0)	0.698 (−0.716–2.111)	0.334
	No	98 (86.0)	Reference	
Dental decay	Yes	35 (30.7)	−0.127 (−1.060–0.805)	0.789
	No	79 (69.3)	Reference	
Sensitive teeth	Yes	15 (13.2)	0.187 (−1.037–1.411)	0.354
	No	99 (86.8)	Reference	
Bleeding gums	Yes	9 (7.9)	−0.263 (−1.974–1.448)	0.139
	No	105 (92.1)	Reference	
Dental checkup every six months	Yes	23 (20.2)	0.677 (−0.392–1.745)	0.676
	No	91 (79.8)	Reference	

Data are presented as numbers (percentages). Reference knowledge category is “low”. β, beta coefficient; 95% CI, 95% confidence interval.

**Table 5:** Generalized linear model analysis of predictors (everyday oral hygiene habits) for a higher total oral health knowledge score.

Characteristics		n (%)	β (95% CI)	*p*
Toothbrush and toothpaste	Yes	111 (97.4)	0.801 (−1.655–3.257)	0.523
	No	3 (2.6)	Reference	
Dental floss	Yes	38 (33.3)	1.578 (0.323–2.834)	0.014 *
	No	76 (66.7)	Reference	
Interdental brush	Yes	26 (22.8)	−0.100 (−1.778–1.579)	0.907
	No	88 (77.2)	Reference	
Tongue cleaner	Yes	28 (24.6)	−1.728 (−3.299–−0.157)	0.031 *
	No	86 (75.4)	Reference	
Mouthwash	Yes	35 (30.7)	0.309 (−0.863–1.480)	0.606
	No	79 (69.3)	Reference	
Breath fresheners	Yes	36 (31.6)	0.922 (−0.294–2.137)	0.137
	No	78 (68.4)	Reference	

Data are presented as numbers (percentages). Reference knowledge category is “low”. β, beta coefficient; 95% CI, 95% confidence interval; * *p* ˂ 0.05.

**Table 6:** Distribution of respondents’ answers to questions about the self-assessment of knowledge about oral health.

Characteristics		n (%)
Self-assessment of personal knowledge about oral health	Very knowledge	14 (12.3)
	Knowledgeable	33 (28.9)
	Somewhat knowledgeable	54 (47.4)
	Not knowledgeable	13 (11.4)
Self-assessment of personal knowledge about oral hygiene	Very knowledge	19 (16.7)
	Knowledgeable	47 (41.2)
	Somewhat knowledgeable	47 (41.2)
	Not knowledgeable	1 (0.9)
Self-assessment of personal knowledge about dental trauma	Very knowledge	6 (5.3)
	Knowledgeable	19 (16.7)
	Somewhat knowledgeable	55 (48.2)
	Not knowledgeable	33 (2.9)
	Not knowledgeable at all	1 (0.9)
The importance of dental trauma knowledge	Very important	8 (7.0)
	Important	51 (44.7)
	Neutral	45 (39.5)
	Low importance	9 (7.9)
	Not at all important	1 (0.9)
The importance of oral health and oral hygiene knowledge	Very important	8 (7.0)
	Important	51 (44.7)
	Neutral	45 (39.5)
	Low importance	9 (7.9)
	Not at all important	1 (0.9)
Source of oral health information	Family and friends	22 (19.3)
	Media	10 (8.8)
	School	19 (16.7)
	Dentist	63 (55.3)
Preferred tools for knowledge management	Online instruction	24 (21.1)
	Brochures/information posters	43 (37.7)
	Lectures	18 (15.8)
	Not interested	29 (25.4)

Data are presented as numbers (percentages).

## Data Availability

The data that support the findings of this study are available upon request from the authors.
